# Unringing the bell: Successful debriefing following a rich false memory study

**DOI:** 10.3758/s13421-024-01524-9

**Published:** 2024-01-29

**Authors:** Ciara M. Greene, Katie M. Ryan, Lisa Ballantyne, Elizabeth Barrett, Conor S. Cowman, Caroline A. Dawson, Charlotte Huston, Julie Maher, Gillian Murphy

**Affiliations:** 1 School of Psychology, University College Dublin, Dublin, Ireland; 2 School of Applied Psychology, University College Cork, Cork, Ireland

**Keywords:** False memory, False belief, Debriefing, Phenomenology

## Abstract

In rich false memory studies, familial informants often provide information to support researchers in planting vivid memories of events that never occurred. The goal of the current study was to assess how effectively we can retract these false memories via debriefing – i.e., to what extent can we put participants back the way we found them? We aimed to establish (1) what proportion of participants would retain a false memory or false belief following debriefing, and (2) whether richer, more detailed memories would be more difficult to retract. Participants (*N* = 123) completed a false memory implantation protocol as part of a replication of the “Lost in the Mall” study (Loftus & Pickrell, *Psychiatric Annals*, *25*, 720-725, 1995). By the end of the protocol, 14% of participants self-reported a memory for the fabricated event, and a further 52% believed it had happened. Participants were then fully debriefed, and memory and belief for the false event were assessed again. In a follow-up assessment 3 days post-debriefing, the false memory rate had dropped to 6% and false belief rates also fell precipitously to 7%. Moreover, virtually all persistent false memories were found to be nonbelieved memories, where participants no longer accepted that the fabricated event had occurred. Richer, more detailed memories were more resistant to correction, but were still mostly retracted. This study provides evidence that participants can be “dehoaxed”, and even very convincing false memories can be retracted.

**Supplementary Information:**

The online version contains supplementary material available at 10.3758/s13421-024-01524-9.

## Introduction

Decades of misinformation research have demonstrated that it is relatively easy to distort or alter an individual’s memory (Frenda et al., 2011; Loftus, 2005). This has clear implications for memory researchers, in terms of the requirement to prevent harm to participants, but also has applications in the real world. Memory distortion has the potential to result in substantial miscarriages of justice, such as when an eyewitness wrongly identifies a suspect as the perpetrator of a crime (Clark & Godfrey, 2009), or when an individual develops a false memory of abuse, for example, during police interviews or in a therapeutic setting (Ceci & Bruck, 1995; Loftus, 2003). Memories implanted in the course of an experimental procedure are typically relatively benign, but may still have unintended consequences for participants. In the absence of a debriefing or memory retraction procedure, these false memories can persist for months or even years (Huffman et al., 1997; London et al., 2009; Zhu et al. 2012). It is therefore important to establish whether implanted false memories can be successfully retracted, both to avoid harm to experimental participants and to safeguard those who come in contact with the legal system.

### Can false memories be retracted?

It is common wisdom that one cannot “unring the bell”: once information has been provided to an individual, it cannot be subsequently wiped from their consciousness. Nevertheless, people can and do revise their judgements regarding whether a particular mental experience should be considered a memory. Several commentators have noted that the identification of a particular mental experience as “remembered” or “believed” is best conceptualised as a metacognitive decision-making process (e.g., Scoboria et al., 2015). The source monitoring framework (Johnson et al., 1993; Mitchell & Johnson, 2000) suggests that memory decisions are made using two monitoring processes: a systematic process, which evaluates whether a memory or mental image is consistent with past experience, and a heuristic process, which considers whether the mental image has the features associated with a real memory. People often spontaneously change their minds regarding their belief in the occurrence of a previously remembered event, for example, if they receive social feedback that the event did not occur as remembered, if the event begins to feel implausible, or if they are provided with other information that contradicts the remembered event (Mazzoni et al., 2010; Scoboria et al., 2015). This new information activates metacognitive processes that lead participants to re-evaluate their previous judgements of the event.

In experimental settings, the contradictory information typically comes in the form of an explicit debriefing or debunking in which participants are informed that previously presented misinformation was not true. Several researchers have investigated the effectiveness of this approach, principally in the context of eyewitness memory and fake news research, and found that misinformation can be successfully retracted and false memories undone. This is typically achieved via a debriefing procedure in which the misinformation is revealed as false (e.g. Greene & Murphy, 2023; Greenspan & Loftus, 2022; McFarland et al., 2007; Murphy et al., 2020). In contrast with this, research into the continued influence effect has demonstrated that misinformation can continue to exert effects even after it has been retracted (see Ecker et al., 2022 for a review). For example, this effect is observed in experimental settings when people continue to believe that a fire described in a fictional newspaper account was caused by arson even after a correction has been issued (Lewandowsky et al., 2012). Similar effects can be observed in real-world contexts, such as when many people continued to believe in the existence of weapons of mass destruction in Iraq despite the absence of any confirmatory evidence (Lewandowsky et al., 2005).

There has been comparatively little investigation of the extent to which rich autobiographical memories can be retracted. One common method of eliciting false autobiographical memories is the false familial informant procedure. In these studies, information provided by an older relative (usually the participant’s parent) is used to create an entirely false event (e.g., Hyman et al., 1995; Loftus & Pickrell, 1995; Wade et al., 2002). The participant is then encouraged to remember details of this event over the course of several interviews. By the end of the procedure, many participants will form a rich and detailed false memory of an event that never happened. False familial informant studies were first devised to provide an “existence proof” (Loftus & Pickrell, 1995, p. 724) for the formation of false memories of childhood experiences, in response to concerns that memories of childhood abuse recovered through therapy might in some cases be false. In a mega-analysis of such studies, Scoboria et al. (2017) reported that nearly half of participants reported either a full or partial false memory for the fabricated event. While many studies of this type report debriefing their participants, few evaluate whether or not that debriefing was effective (though see Otgaar et al., 2013, in which the majority of participants retracted their false memory claims following a detailed debriefing).

In a recent study, Oeberst et al. (2021) evaluated a method for reversing false autobiographical memories implanted during an experimental task. Participants completed a familial informant procedure in which their parents provided details of plausible but false events from the participant’s childhood. The participant then attended three interviews, and were asked about each of the false events under conditions of minimal suggestion (in which researchers simply established a rapport with participants and informed them that the childhood events had been provided by their parents) or massive suggestion (in which researchers also employed a range of highly suggestive techniques to elicit memories, including guided imagery and context reinstatement, verbal reinforcement of any details provided by the participant, and active encouragement of participants to try harder to come up with a memory). Interview transcripts were then coded for the presence of false memories using the coding scheme provided by Scoboria et al. (2017). The memory reversal procedure took place at the end of the third interview, and incorporated two elements: source sensitisation, in which participants were reminded that memories can sometimes be based on elements other than our own recollections, such as photographs or the descriptions of family members, and false memory sensitisation, a form of psychoeducation in which the reconstructive nature of memory was explained to participants. By the end of the sensitisation procedure, 15% of participants in the minimal suggestion condition and 23% of participants in the maximal suggestion condition continued to report a false memory, down from 27% and 56%, respectively, at the end of the third interview. A substantial proportion of participants did, however, continue to believe that the event had occurred, with approximately 60% of participants coded as accepting the false memory in the massive suggestion condition. This was reduced substantially by a 1-year follow-up, where approximately 25% of participants continued to accept or remember the false event. Thus, while the debriefing procedure was effective for the majority of participants, there were some who continued to have persistent false memories or beliefs of the fabricated event. There are several potential explanations for this, with different consequences for participants and researchers.

First, participants may have been unwilling to identify events as false immediately following the sensitisation procedure due to trust in their parents’ narrative (Brewin & Andrews, 2017) or rapport with the researcher (Porter et al., 2000). This does not, however, explain the participants who reported a persistent memory or belief after 1 year, by which time they had been fully debriefed and had had the opportunity to discuss the study with family members.

Second, the persistent memories may have been particularly “sticky” (Lewandowsky et al., 2012) – that is, they may have had features that rendered them especially resistant to correction. The subjective experience of recalling an event (true or false) is closely linked with the phenomenology of the memory; that is, its perceptual and emotional characteristics (Scorboria et al., 2014). In general, real memories tend to be phenomenologically richer and more detailed than false memories (Kealy & Arbuthnott, 2003; Ost et al., 2002). Moreover, if a person produces a mental image rich in phenomenological features, such as colour or sound, they are more likely to believe the event happened (Bernstein et al., 2002). The source monitoring framework predicts that a mental representation of a false event that seems especially plausible (i.e., consistent with the participant’s general recollections of their childhood) or especially rich in detail is likely to be mistaken for a real memory in the first place and may also be more difficult to retract.

Third, as Oeberst et al. note, the events used in their study were designed to be very plausible (e.g., getting lost, running away, or being involved in an accident). It is possible that some version of the fabricated events actually did occur for some participants, and that their informants had simply forgotten. These participants would then be retrieving a real memory (rather than constructing a false one) and might be resistant to the idea that their memories were not true.

Finally, we must consider the possibility that some participants retained a memory of the event while no longer believing in its occurrence – a so-called “nonbelieved memory” (Mazzoni et al., 2010).

### Nonbelieved memories

False memory research makes the distinction between *remembering* an event and *believing* that the event happened (Mazzoni et al., 2001; Scoboria et al., 2014). Most memory coding schemes characterise false memories as involving both recollection and belief in occurrence (Bernstein et al., 2015; Scoboria et al., 2017), and thereby distinguish between memories of the fabricated event and mere acceptance. Efforts to retract false memories are based in part on the idea that belief and memory are nested, such that belief in the occurrence of the event is a necessary precursor to the formation of a memory for the event. Indeed, belief in occurrence has been conceptualised as a metacognitive cue in the source monitoring process, providing supporting evidence that a memory represents a real event (Clark et al., 2012). In this framework, removing the belief in the event’s occurrence by means of a debriefing should also remove the foundation supporting the false memory, causing it to collapse. This notion is challenged by the existence of nonbelieved memories, whereby people remember an event without believing it took place. Piaget (1951) provides a striking example of this, describing a memory of an event from his childhood in which he had nearly been kidnapped but his nanny fought off the attacker. As an adult, Piaget learned that this event had never actually happened – the nanny had made up the story – but he still retained a vivid mental image of the attack. A more quotidian example may be found in the memories of many people who “remember” seeing Santa Claus come down the chimney or hearing his reindeer on the roof on Christmas Eve (Mazzoni et al., 2010). As adults, these people no longer believe in Santa, and do not believe they really saw him in their living room. Nevertheless, they can access this “memory” at will.

Nonbelieved memories may be created in a few ways. In the first place, there are memories that the participant has never believed in – for example, those obtained in imagination inflation studies where the participant is encouraged to form a detailed mental image of a fictional event, such as winning a stuffed animal at a carnival or breaking a window with their hand (Garry et al., 1996). By some definitions, these participants might be considered to be remembering the event, since they can describe it in considerable detail, but the crucial element of belief in occurrence is absent. Individuals may also lose belief in the memory organically when they encounter new and contradictory information (e.g., when Piaget’s nanny admitted that she had invented the story of the attempted kidnapping, or when people learn that there is no Santa Claus). Of more relevance to the present study, there is ample evidence that the debriefing process itself can result in nonbelieved memories for implanted false memories (Otgaar et al., 2014; Scoboria et al., 2015). These memories may still receive high ratings of phenomenological characteristics despite much-reduced belief in occurrence (Clark et al., 2012). Combined with the possibility that participants may be unwilling to explicitly reject an event that has been provided to them by a trusted family member and respected researcher (Brewin & Andrews, 2017; Oeberst et al., 2021), it is possible that at least some persistent false memories following debriefing may in fact be nonbelieved memories. This presents significantly fewer ethical concerns than the alternative, given the strong evidence that it is belief in an event, rather than memory per se that determines behaviour (Bernstein et al., 2015). Nevertheless, the ethical implications of implanting false memories in the minds of unwitting participants are particularly important to consider when discussing the effectiveness of debriefing procedures.

### Ethical issues in false memory studies

By its very nature, misinformation research usually requires deception, first as to the true purpose of the study, and second as to the content of the misleading materials themselves. This presents a clear ethical dilemma: informed consent is a cornerstone of human subjects’ research and is threatened by deceptive practices, since participants are typically not provided with sufficient information about the true nature of the study to make a fully informed decision as to their participation (Benham, 2008; Miller et al., 2008). Deception may also run counter to the ethical principle of beneficence; that is, the need to benefit those we work with and strive to do no harm (American Psychological Association, 2017). Deception has the potential to cause real-world harm to the participant or society if they accept misinformation presented to them in the course of a study as true (e.g., if a participant came to believe that vaccines are dangerous or that an elected official is untrustworthy). Deception can also cause emotional distress to the participant or damage their trust in science if they feel foolish or betrayed upon learning that they have been duped.

In many cases, the misinformation provided as part of false memory studies is relatively benign. In eyewitness memory paradigms, for example, participants may be misled regarding aspects of a scene, and thereby come to believe that a car was blue rather than green (Loftus, 1977), or that they saw a yield sign rather than a stop sign (Loftus et al., 1978). In other cases, the misinformation may be more insidious and may lead a participant to question aspects of their own life or behaviour. False memory implantation studies may pose a particular ethical risk if the memories are persistent or have the potential to influence the participant’s future behaviour or psychological wellbeing. For example, it is conceivable that coming to remember that your parent lost you in a shopping centre (as in Loftus & Pickrell, 1995) might damage your trust in that parent, or that believing that you committed a crime as a teenager and were arrested (as in Shaw & Porter, 2015) might elicit feelings of fear or trauma. Moreover, evidence suggests that false memories can have behavioural consequences – for example, a body of research has found that inducing false memories of getting sick after eating a particular food can reduce willingness to eat that food in future (see Bernstein et al., 2011, for a review). Despite these concerns, studies have reported that participants themselves are happy to take part in deceptive research, understand its necessity, and report enjoying the experience (Boynton et al., 2013; Murphy et al., 2020; Murphy, Maher et al., 2023b).

In order to mitigate the risks described above, most psychological associations (including the British Psychological Society, American Psychological Association, and Psychological Society of Ireland) mandate a debriefing following deceptive research. The primary goals of debriefing are typically (1) to “dehoax” the participant (Holmes, 1976) and ensure that they have an accurate understanding of the facts, and (2) to undo any harm that might be caused by the deception. In the case of false memory research, this includes retracting the inaccurate information provided to participants in the course of the experiment. Implicit in these goals is the assumption that debriefing works: that we can effectively retract misinformation and undo false memories. This is, however, an empirical question that has to date been understudied.

### The present study

This study aimed to investigate the effectiveness of a full debriefing procedure for retracting false autobiographical memories and beliefs. In a recently published paper, we (Murphy, Dawson et al., 2023a) reported a successful replication of the landmark Lost in the Mall study, originally described by Loftus and Pickrell (1995). In this replication, participants were recruited along with an adult familial informant (usually the participant’s mother). The informant described various events from the participant’s childhood and provided details that were used to create a fictional event, in which the participant got lost in a shopping centre at the age of five. Participants were then interviewed via video call on two separate occasions. A coding scheme was applied to interview transcripts to determine the presence or absence of false memories and beliefs. In an extension of the original study, we also asked participants to self-report whether they remembered or believed each of the events, answering simply “yes” or “no”. The goal here was to allow participants to have the final say regarding whether or not they had experienced a false memory, given ongoing discussion about the validity of different memory coding schemes (Shaw, 2018; Wade et al., 2018).

Participants were fully debriefed at the end of the second interview, over video call, and the nature and provenance of the false event was explained. The aim of the present paper was to ask whether the debriefing process effectively retracted false memories and beliefs, when assessed immediately and after a delay. The debriefing procedure was based on recommendations listed in Greene et al. (2022), and followed the guidelines for debriefing provided by the Psychological Society of Ireland, which are in line with those provided by other national and international bodies. Specifically, the code requires researchers to “clarify the real nature of and rationale for the study, and seek to remove any misconceptions and re-establish trust” (Psychological Society of Ireland, 2019, p. 17). Our goal was to evaluate the effectiveness of a typical debriefing; thus, the procedure did not include any special features, such as the source sensitisation and false memory sensitisation described by Oeberst et al. (2021). The debriefing was designed to match current best practice in the field (Murphy & Greene, 2023); it is, however, hard to know how frequently this approach is actually employed, as misinformation researchers rarely report details of their debriefing procedures in their papers (Greene et al., 2022).

A secondary aim of the present study was to explore the role of phenomenology of a false memory in the debriefing process, with the expectation that particularly vivid memories might be “stickier” and thus especially difficult to retract. Previous work on believed and nonbelieved memories has suggested that distinct features of the mental experience underlie belief and recollection, with perceptual and emotional features predicting recollection of the event but not belief in occurrence, while belief is predicted by perceived plausibility of the event (Scoboria et al., 2014). Phenomenological characteristics of a memory, including its clarity, the presence of audiovisual elements and its tendency to trigger other memories or produce an emotional reaction have previously been reported to distinguish between true and false memories (Marche et al., 2010). We aimed to establish whether the features of the implanted memory would predict initial belief in and recollection of the fabricated event, and whether these features would predict continued memory or belief in recollection after the misinformation had been retracted.

We addressed the following preregistered research questions:

1. What proportion of participants retain a false memory, false belief, or nonbelieved memory regarding an implanted memory following a full debriefing procedure?
2. Does the phenomenology of the false memory predict the effectiveness of the debriefing? Specifically, we asked whether memories that were reported as more rich or detailed would be more difficult to retract, resulting in a higher rate of persistent false memories/beliefs after debriefing.

## Method

### Preregistration

The full Lost in the Mall replication project was preregistered on the Open Science Framework at https://osf.io/krfpu/. This was a large project incorporating multiple student researchers, each of whom had a distinct research question. In the interests of transparency, we included all research questions in the preregistration document. The research questions underlying the present paper were pre-planned as a separate paper and are listed under RQ3, pages 19–22. The post-debriefing measures described here are not reported in any other paper.

### Participants

The present study focusses on participants who completed the Lost in the Mall paradigm as part of the replication study described in Murphy, Dawson et al. (2023a). We preregistered a target sample of between 100 and 150 participants, recruited before a preregistered stopping date. The sample size for the replication study was not informed by power calculations, as the original Loftus and Pickrell (1995) study did not include any inferential statistics. We identified 150 participants as the maximum practically achievable sample size, given the time-consuming and labour-intensive method of data collection. All participants who completed the replication study were included in the present analyses. The final sample included 123 participants (81 female, 42 male), ranging in age from 18 to 57 years (M = 25.49 years, SD = 6.74). Participants were recruited primarily via the personal networks of student researchers at University College Dublin and University College Cork. The researchers avoided recruiting their fellow psychology students as they may have been aware of the original study, which is frequently discussed in undergraduate psychology lectures.

### Materials and procedure

**Fig. 1:**
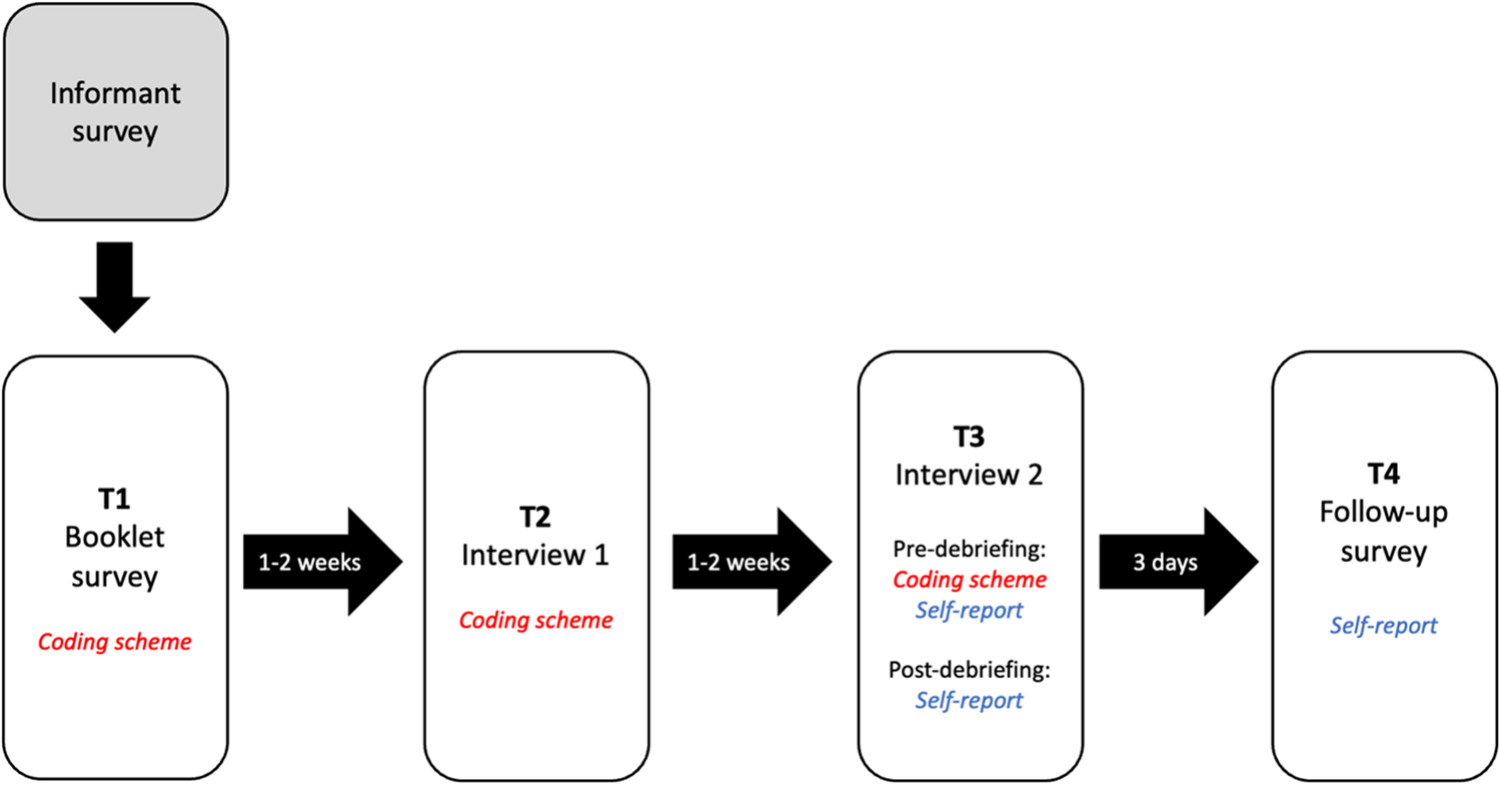
Timeline of false memory implantation, debriefing and follow-up. The method used to categorise memories at each stage is indicated in italics

See Fig. 1 for a timeline of the study. As detailed in Murphy, Dawson et al. (2023a), familial informants (almost all of whom were the participants’ mothers) completed a survey in which they provided information about the participant’s childhood. Informants were asked to describe some events from when the participants was about 5 years old, and to provide details regarding a typical shopping trip from the same period (e.g., where did the family usually shop, who would have gone along on these trips etc.). This information was used to create the initial survey that was sent to participants via email. We refer to this as the “booklet” survey, for comparison with the original Loftus and Pickrell study in which a pen-and-paper booklet was used. In this survey, participants were provided with a brief summary of four childhood events. Three of these were true events, provided by the informants. The final event was the false “lost in the mall” story. This story was created from a template and completed with details provided by the informant regarding a typical shopping trip when the participant was about 5 years old. An example of the story provided to one participant is given below:

*You, your mum, your brother Christopher, Sam and Neill all went to Toy Master. You were 5 years old at the time, you ran ahead into the shop and went to the Lego section to pick out your favourite set. When you went to look for your mum you couldn't find her. You became very upset when you realised you were lost and starting crying. An elderly lady found you and helped you return to your mum. Afterwards, you all went to the Lego aisle and you picked out a car set.*

Participants were encouraged to write as much as they could remember regarding each event in an open text box. The false event always appeared in the third position in the booklet survey, in line with the original study.

After completing the booklet survey, participants were contacted to schedule two interviews, which took place over Zoom or MS Teams approximately 1–2 weeks apart. During each interview, which lasted for approximately 20–30 min, participants were asked to recall as much as they could remember about each of the four events. Interviewers remained friendly, but pushed participants to recall more detail using a selection of prompts (e.g., “Can you remember anything more about that?”; “Think about it a bit more and it might come back to you”). If participants struggled to recall details, the interviewers provided small details from the original story to jog their memory (“I think you were with your aunt, is that right?”). The interview schedule and transcripts may be found online at https://osf.io/krfpu/.

During the second interview, participants were asked to rate the phenomenological features of each memory on six dimensions, using a 10-point Likert scale. Some questions were adapted from the original study (Loftus & Pickrell, 1995), and others from Marche et al. (2010), who used a modified version of the Memory Characteristics Questionnaire (Johnson et al., 1988). The questions used in the present study were as follows:

1. Clarity: “How clear is this memory?” *(1 = not at all clear, 10 – very clear)*
2. Confidence in future memory: “How confident are you that, if given more time, you’d be able to remember the event in more detail?” *(1 = not at all confident, 10 = very confident)*
3. Sound: “My memory for this event involves sound” *(1 = definitely not true, 10 = definitely true)*
4. Evokes a feeling/reaction: “This memory evokes a feeling/reaction from me; i.e., this memory triggers a thought, emotion, or image” *(1 = definitely not true, 10 = definitely true).*
5. Triggers memories: “This memory has made me think of another event/memory from my life” *(1 = definitely not true, 10 = definitely true).*
6. Ease of access: “I can easily access this memory without much effort”* (1 = definitely not true, 10 = definitely true).*

Participants were then asked if they remembered each event one last time, and provided a simple yes/no response. If the participant did not remember the event, they were asked if they believed the event happened. We did not ask participants who did report a memory whether they also believed in the event, as at this stage in the procedure participants had not yet been debriefed and we wanted to avoid arousing suspicions about whether the events were true, particularly given that the fake “lost in the mall” event was the third event to be discussed. The participant was then asked to rate how likely they would be to testify in court that each event really happened, on a scale of 1–10, where 1 indicated “not at all likely”, and 10 indicated “very likely to testify”. This item was not relevant to the questions addressed in the present manuscript. Participants were then debriefed.

### Debriefing

As part of the debriefing process, participants were first informed that one of the events from their childhood had not really happened, and were asked to try to identify the fake event. The “lost in the mall” event was then revealed to have been fabricated by the researchers. The interviewer carefully explained that this event had not happened, but had been made up by the research team (not by the participant’s informant). They then described the purpose of the study and the reason for deception. The original Loftus and Pickrell study was explained, and participants were educated about memory distortion. Particular care was taken during the debriefing process, and interviewers ensured a slow pace and understanding tone throughout. The script that interviewers followed for the debriefing may be found in the interview schedule document at https://osf.io/krfpu/. Participants were asked to re-confirm consent for the use of their data, now that they were fully informed of the purpose of the study. No participants withdrew consent at this stage.

Interviewers then explained the difference between a memory and a belief, emphasising that these may exist independently: “Sometimes there are events that we believe happened, but we don’t remember happening – for example, I believe I was vaccinated as a baby, but I don’t remember it happening. On the other hand, sometimes we have memories of events that we don’t really believe happened. For example, many people remember seeing Santa Claus coming down the chimney when they were a child, but as an adult they don’t believe it really happened”. Participants were then asked to reflect on the fake “lost in the mall” event, and answer yes or no to the questions, “Do you currently remember the event happening?” and “Do you currently believe the event happened?”. If participants indicated that they still believed in the false event, they were asked to explain why. If participants did not readily provide an answer, they were offered three possible reasons: “I think my [informant] was lying and this really did happen”; “I think my [informant] was mistaken and this really did happen”, and “I think you’re lying to me now in saying that it didn’t happen”.

The interviewer then explained that they would be sending a follow-up survey in the following days. The participant was thanked for their time and the interview was concluded.

### Follow-up surveys

Follow-up surveys were sent via email three days after the second interview. This interval was chosen by the research team, as there was no follow-up in the original paper. The email containing the follow-up survey came not from the interviewer, but from one of the principal investigators who had not been involved in the recruitment or interviewing of participants, in order to reduce socially desirable responding arising from the rapport that the participant may have developed with the interviewer over the course of the two interviews.

The survey began by repeating the explanation about the difference between memory and belief provided during the second interview, and then continued, “Thinking about getting lost in a shopping centre, as described in the interview, do you currently believe that the event happened?” Participants could select from the options, “Yes, I believe this happened”, “No, I don’t believe this happened” and “I'm not sure/I don't know how to answer this question”. Participants were then asked, “Do you currently remember that the event happened?” and could select from the options, “Yes, I remember this happening”, “No, I don’t remember this happening”, and “I'm not sure/I don't know how to answer this question”. Participants who answered “yes” to the memory question were also asked to select the best description of their memory of the event from the following options: “I have a very clear, detailed memory of this event”; “I have a partial memory of this event (where some aspects are clear and detailed)”; “I have a vague memory of this event”; “I do not remember this event”.

Participants were then asked to answer some questions regarding their relationship with their informant, which are not relevant to the present study, and to reflect on the ethics of deceptive research. Data relating to the ethics questions are described in Murphy, Maher et al. (2023b). At the end of the follow-up survey, participants were once again given the option to retract their data, now that they were fully informed about the study aims. No participants chose to do so.

### Ethics

Ethical approval for the full project, including all research questions and follow-up assessments, was obtained from the psychology research ethics committee at University College Cork.

### Data coding

The interview transcripts were coded by four pairs of independent coders according to a manual (see Online Supplemental Materials (OSM)). Disagreements were resolved by a third coder who was one of the principal investigators (GC or GM). Coders first transcribed ratings of phenomenological features of each memory provided during the interview (kappa = 0.97), and participants’ self-reports regarding whether they remembered or believed each event (kappa = 0.93). The coders then applied an adapted version of the original Loftus and Pickrell (1995) coding scheme to the participants’ description of each event, and categorised them as representing a full memory, partial memory or no memory (kappa = 0.60). The full coding scheme can be found in the OSM, and a detailed discussion of how the scheme was adapted can be found in the replication paper, Murphy, Dawson et al. (2023a).

## Results

### Classification of false memories and beliefs

Using the adapted Loftus and Pickrell (1995) coding scheme, 35% of participants were coded as having formed a false memory for the fake event by the end of the second interview. Details of these analyses may be found in Murphy, Dawson et al. (2023a), and the full dataset is available online at https://osf.io/krfpu/.

Self-reported memory and belief were also recorded at three timepoints (during Interview 2 both before and after debriefing, and in the follow-up survey three days later; see Fig. 1). In line with our preregistration, the analyses in the present paper focus on the self-report measures of memory and belief, in order to allow direct comparison between the pre-debriefing, post-debriefing and follow-up timepoints. Using this more conservative approach, 17 participants (13.8%) self-reported a memory of the fabricated event by the end of Interview 2, and a further 64 (52%) reported believing that the event had happened. Thus, about two-thirds of the sample fell for the misinformation.

### Effectiveness of debriefing

**Fig. 2:**
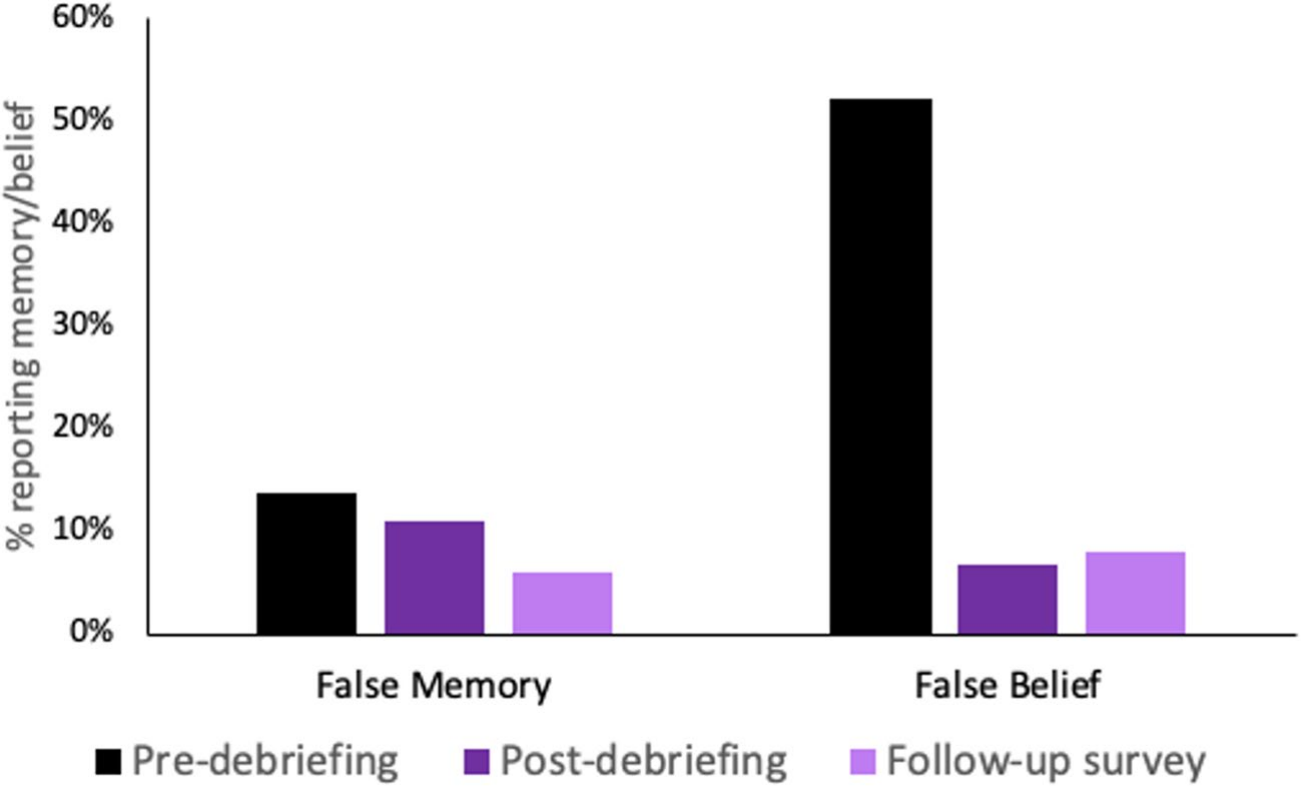
Percentage of participants self-reporting false memories and false beliefs at each timepoint

Immediately following the debriefing, 13 participants (11%) reported a false memory, representing a 20.2% reduction from the 13.8% of participants who reported a memory at the pre-debriefing assessment. Just eight participants (6.8%) reported believing that the event had happened, representing an 87% reduction in false belief relative to the 52% of participants who reported a belief at the pre-debriefing assessment. Three days later, 101 participants completed the follow-up questionnaire. The proportion of participants who completed the follow-up questionnaire did not differ as a function of whether or not participants had initially reported a false memory (*X*^2^ (1) = 1.78, p = .18) or false belief (*X*^2^ (1) = 0.02, p = .89). At follow-up, the false memory rate was reduced to 6% (*n* = 6; i.e., a 57% reduction from the pre-debriefing assessment) and the false belief rate was 8% (*n* = 8, an 85% reduction from the pre-debriefing assessment). Thus, the majority of false memories and beliefs were retracted following debriefing, but a small number persisted. While the false belief rate fell very quickly (immediately following debriefing), retraction of false memories took a little longer, and a substantial decrease was not observed until the follow-up three days later. See Fig. 2 for an illustration.

Table 1 displays a crosstabulation of persistent memories and beliefs for the false event. Among participants who reported still remembering the “lost in the mall” event following the debriefing, less than a third reported also believing that the event really happened. In other words, two-thirds of the persistent false memories were in fact nonbelieved memories. Participants who reported still believing in the fake event following debriefing were asked to give a reason for their answer, selecting from the options “I think my [informant] was lying and this really did happen”; “I think my [informant] was mistaken and this really did happen”, and “I think you’re lying to me now in saying that it didn’t happen”. Of the eight participants who reported a persistent belief, four indicated that they believed their informant was mistaken. No participants selected the options indicating that their informant or the experimenter were lying. Three participants’ responses were coded as ‘other’; these participants gave detailed answers regarding the plausibility of the event (e.g., Participant 313: “it's just the whole scene, it makes sense. It's the type of thing that would have happened”). Several participants reflected on their own memory processes. For example, one participant gave the following response:

“It's definitely having, I think, this whole conversation… I’ve put so much time into this sort of construction. It's starting… like it does feel like you're talking about a memory more than anything else, it does. Yeah, yeah. It definitely... like putting this much time into a memory that you were thinking was real, just… that's probably why my gut feeling is telling me yes. It's because we've spent so much time talking about this memory that you kind of feel like it is... like it is real” (Participant 238).

**Table 1:** Cross-tabulation of number and percentage of participants reporting memories and beliefs for the false event following debriefing

	Interview 2 post-debriefing		Follow-up survey	
	Belief	No Belief	Belief	No Belief
Memory	4 (31%)	9 (69%)	2 (33%)	4 (67%)
No memory	4 (4%)	101 (96%)	6 (6%)	89 (94%)

One participant was not asked this question as, despite having given a “yes” response to both the memory and belief questions, she later clarified that she believed she had in fact gotten lost on another, similar occasion.

By the follow-up survey, just two participants reported a false memory of the event that they actually believed had happened. In both of these cases, the participant appeared to believe that they had gotten lost under different circumstances than those described in the false mall event, and were confused that the details provided by the interviewer differed from their memory. It is therefore possible that these two participants were actually lost as children, and that their informants were mistaken.

During the follow-up survey, participants were asked, “Thinking back, do you think you exaggerated or ‘went along with’ the interviewer during the study, when discussing getting lost in a shopping centre? (i.e., said things you knew or suspected were untrue)”. Participants responded on a 5-point Likert scale, with 5% of participants saying “definitely yes”, 12% “probably yes”, 7% “might or might not”, 39% “probably not”, and 37% “definitely not”. Thus, the majority of participants indicated that their responses were not influenced by socially desirable responding. Participants were also asked whether they had engaged in any discussions with their informant or anyone else over the course of the study. The vast majority of participants – 98% – denied discussing the event with their informant. Just two participants reported discussing the event with their informant, but the discussions were described as very brief (“I told my mother I could not wait to discuss the events spoken about after the study, my mam responded telling me it was all events we have talked about. I remember being confused because I did not recall two of the events but I did not ask anymore”; “Just the zoo memory for a few seconds but it didn't help me remember anything else”). When asked if they had discussed the event with anyone else, 80% of participants said no. An additional 15% reported discussing the event with someone not familiar with their childhood (e.g., a romantic partner or a friend they met as an adult). One participant reported discussing the event with a person named in the story, and two discussed the event with someone else familiar with their childhood. Overall, these results give us confidence that the pattern of results was not influenced by socially desirable responding or by discussion of the events with the informant or other people with knowledge of the participants’ real childhood experiences.

### Phenomenology of false memories

During Interview 2, participants rated each memory on six features: clarity, confidence in future memory, sound, evokes a feeling/reaction, triggers memories, and ease of access. Our preregistration called for logistic regression analyses to identify the features that predicted a persistent false memory or belief following debriefing. Interested readers may find the preregistered analyses in the OSM (Tables S-1 and S-2). However, as seen in Table 2, there was substantial multicollinearity among the predictor variables, which rendered the regression analyses unreliable (resulting, for example, in flipped signs for individual predictors).

**Table 2:** Descriptive statistics and correlation coefficients for phenomenological features of the false event

	Clarity	Confidence	Sound	Evokes a feeling/reaction	Triggers memories	Ease of access
Clarity	-					
Confidence in future memory	.65**	-				
Sound	.56**	.46**	-			
Evokes a feeling/reaction	.48**	.45**	.26**	-		
Triggers memories	-0.02	0.12	-.21*	0.16	-	
Ease of access	.87**	.59**	.53**	.51**	-0.05	-
*Mean*	2.15	2.20	1.82	3.56	5.19	1.95
*SD*	1.68	1.70	1.58	2.42	2.97	1.75

* Correlation is significant at the 0.05 level (two-tailed)

** Correlation is significant at the 0.01 level (two-tailed)

Given the strong linear relationships between the phenomenology measures, and the fact that these variables were conceptualised as different aspects of the same underlying construct – the richness of the false memory – we instead conducted a set of multivariate analyses of variance (MANOVAs) in which the six features were entered as dependent variables, and the presence (or absence) of a false memory/false belief at each point of assessment was entered as the independent variable. For each outcome, this allowed assessment of the overall richness of the memory, defined as a combination of the dependent variables, and a separate examination of the univariate effect of each of the six features. The available sample size provided 80% power to detect a small to moderate multivariate effect, *f*^2^ = 0.11.

We first compared ratings for the fake event between participants who reported a false memory at the end of the second interview (before debriefing) and those who did not. A significant multivariate effect was observed: overall, memories were rated as richer than non-memories. Multivariate analyses along with descriptive statistics for the average (composite) of the six phenomenological features may be seen in Table 3. Univariate comparisons for each of the six features may be seen in the OSM, Table S-3. False memories were rated significantly higher than non-memories on all features except “triggers memories”, which did not differ between groups. In line with our preregistration, we next expanded the definition of a false memory to include false belief. In this case, the multivariate effect was not significant (see Table 3). Examination of the univariate effects in Table S-3 shows that participants who reported a memory or belief rated the fake event as significantly higher in clarity, confidence, sound, and ease of access than those who did not. There was no significant difference in the extent to which the memory evoked a feeling/reaction or triggered further memories between these groups.

**Table 3:** Descriptive statistics of the composite ratings of phenomenological richness and multivariate analysis of all six phenomenological features, compared between those who reported a memory (or belief) for the fake event at each time point, and those who did not

	False memory			No false memory			
	N	M	SD	N	M	SD	Multivariate comparison
Pre-debriefing	17	4.42	1.59	106	2.55	1.06	F(6, 116) = 16.89, p < .001, η_p_^2^ = .83
Post-debriefing	13	3.86	1.13	105	2.72	1.29	F(6, 111) = 5.23, p < .001, η_p_^2^ = .22
Follow-up survey	6	4.08	1.35	95	2.68	1.16	F(6, 94) = 3.54, p = .003, η_p_^2^ = .18
	False memory OR belief			Neither false memory NOR belief			
	N	M	SD	N	M	SD	
Pre-debriefing	81	3.04	1.42	42	2.36	0.92	F(6, 116) = 1.80, p = .105, η_p_^2^ = .08
Post-debriefing	17	3.71	1.18	101	2.70	1.30	F(6, 111) = 3.90, p = .001, η_p_^2^ = .79
Follow-up survey	12	3.28	1.28	89	2.70	1.20	F(6, 94) = 1.52, p = .18, η_p_^2^ = .09

We next asked whether these phenomenological features (provided at the end of the second interview, prior to debriefing) would influence participants’ tendency to retain a false memory or false belief immediately after being debriefed. The multivariate analysis in Table 3 indicates that participants who reported richer memories of the fake event were more likely to continue to report a false memory or a false memory or belief post-debriefing. Univariate analyses indicated that, regardless of which categorisation was used, participants who continued to accept the false memory rated the fake event higher for clarity, confidence, sound and ease of access, with no difference in the degree to which the event evoked a feeling/reaction or triggered other memories (see Table S-4, OSM).

Turning to the assessment of false memory in the follow-up survey, there was a significant multivariate effect of group, such that the few participants who continued to report a false memory gave higher overall ratings of the richness of the memory than participants who did not. This multivariate effect was not observed when comparing participants who reported either a false memory or belief against those who didn’t. Examination of the univariate effects (Table S-5, OSM) indicates that, regardless of how memories and beliefs were grouped, participants who continued to accept the fake event at the follow-up had experienced memories that were clearer and easier to access.

## Discussion

The results of this study demonstrate that a full debriefing can effectively retract false autobiographical memories and beliefs. Sixty-eight percent of participants reported a memory or belief for the fabricated event by the end of the memory implantation procedure, but this was reduced to 14% immediately following the debriefing, and to just 12% by the follow-up survey 3 days later. Moreover, all but two of the memories that persisted through to the follow-up session appeared to be nonbelieved memories. This is in line with previous findings that suggested a false belief is easier to implant and remove than a false memory (Otgaar et al., 2013).

The debriefing process was successful at immediately reducing false belief, but false memories took longer to be retracted, with a steeper decline between the post-debriefing and follow-up assessments. This is consistent with previous findings that debriefing leads to a stronger decline in false belief than false memory (Clark et al., 2012), but may also be attributed to a need for participants to take the time to process the deception and reconsider the memories retrieved during the study. Alternatively, there may have been some social desirability at play, where participants were reluctant to reverse themselves and admit to no longer remembering something they had described in detail to the interviewer mere minutes previously. The delay may also have allowed participants to discuss the childhood events with their informant and receive reassurance from a trusted source that the event did not take place.

False memories can have grave consequences, as demonstrated by the “memory wars” of the 1980s and 1990s, during which a number of people formed novel memories of abuse following suggestive questioning or psychotherapy (see Schacter, 1995; Loftus 2004, for overviews of this period). People are more likely to act on their memories if they believe the events contained in those memories to be true (Bernstein et al., 2015). The retraction of a false belief may therefore be more important than (likely impossible) attempts to remove all traces of the subjective memory experience. The fact that the remaining memories in the present study were mostly nonbelieved is therefore reassuring from an ethical perspective: it seems highly unlikely that someone would take actions that were detrimental to their life or well-being on the basis of a memory that the person does not believe to be true.

In the case of the small number of persistent, believed memories, we speculate (but cannot prove) that these participants may have actually been lost at some point in their childhood, resulting in a “false” memory that is, in some respects, true. We should not make the mistake of assuming that the informants were infallible: the parents of the participants are just as likely to suffer from memory distortions as their children, and may have forgotten or been confused about specific events. In support of this, a number of informants told us in a follow-up survey that, in retrospect, they were concerned that they may have mixed up the participant with another of their children; for example, one informant wrote, *“I did wonder about the fact that as I have four children it would be easy to muddle up the various memories, for example, which child did that happen to”.* See Murphy, Maher et al. (2023b) for details and a discussion of the informant follow-up questionnaire.

Participants were more likely to report a false memory if their subjective experience was richer: participants who reported a false memory gave their memories of the fake event higher ratings of all measures of phenomenology, with the exception of the tendency of the memory to trigger other memories, which did not differ between groups. This is perhaps unsurprising, given that clarity/vividness has been conceptualised as the quintessential feature of autobiographical memory (Greenberg & Rubin, 2003). Richer memories were also more resistant to correction: participants who continued to report a false memory or belief following the debriefing reported having memories of the fake event that were clearer, easier to access, remembered with greater confidence, and more likely to include sound. These memories were also associated with a stronger tendency to evoke an emotional reaction, though this did not reach statistical significance. Overall, persistent false memories were associated with a greater degree of richness and phenomenological detail than descriptions of the fake event by participants who no longer reported a memory. Thus, our hypothesis was largely supported: richer, more detailed memories were indeed “stickier” and harder to retract than less vivid recollections.

Some limitations of the study ought to be considered. As noted above, a very small number of participants continued to report a false memory or belief by the time the follow-up survey was administered 3 days later. The unbalanced sample at the post-debriefing and follow-up assessments was a direct consequence of the success of the debriefing process; however, it is worth noting that this may have consequences for the power of the analyses as outliers in the smaller group might have had an undue impact on the group mean, biasing results. As always, replication is required to validate the findings reported here. There may also have been some selection bias in the completion of the follow-up survey; just 101 of the original 123 participants responded. Attrition rates did not differ between those who had formed a false memory/false belief and those who did not. It is, however, possible that those who had a more negative experience or felt more aggrieved by the deception may have been less likely to complete the follow-up survey, and this may have affected our findings. Future research might also conduct repeated follow-ups to see how false memories and beliefs may change over time following debriefing. In the current study we felt this was not possible, as participants had already contributed so much time and were not compensated in any way, but previous studies have established that false memories and beliefs can persist for over 12 months (Oeberst et al., 2021), and so this should be examined further.

This study provides practical insight for false memory researchers. We recommend that a slow, thorough debriefing is offered to participants who take part in rich false memory studies. As we have discussed elsewhere (Murphy, Maher et al., 2023b), this should include debriefing the informants who unwittingly helped to deceive their relative. We also recommend specific ethical practices, including following up with participants to check that the debriefing was effective, and offering participants the chance to withdraw their data once they learn the true purpose of the study. A recent scoping review of misinformation research found that these practices are vanishingly rare – less than 1% of papers from 2016–2021 reported engaging in either practice, though when contacted, some authors reported they did engage in these practices but did not include them in their paper (Greene et al., 2022). The current study suggests that standard ethical practices in false memory research are likely quite effective, but we would stress that they must be implemented carefully and reported rigorously.

The present study evaluated the effectiveness of a standard debriefing procedure and did not experimentally manipulate debriefing conditions. Thus, we make no claims about the relative effectiveness of this approach compared to other debriefing procedures. As the majority of misinformation papers to date have not reported details of their debriefing procedures, our ability to make comparisons with the literature is limited. We recommend that future research should address this question by experimentally evaluating the impact of different components of debriefing on subsequent memory and belief, with the goal of identifying the most effective approach for any given study.

We also note some constraints on the generality of the conclusions reported here. The present study evaluated the effectiveness of debriefing in a particular context, and in relation to a single, fairly plausible and non-traumatic event. The metacognitive processes that re-evaluate judgements regarding memory and belief in the light of new information (Scoboria et al., 2015) may operate differently with an event that is either more or less plausible or that produces more intense emotional reactions (as might be expected for memory of a traumatic incident). The false memories in this study were also implanted using a very specific false familial informant paradigm, in which events were described as originating with a parent but were presented to the participant by an unfamiliar researcher. A different social context, in which either the misinformation or the correction were provided by a person with whom the participant had a social relationship, might also change the dynamics of the debriefing process.

In summary, this study provides convincing evidence that participants can be “dehoaxed” and implanted false memories can be retracted. If a careful debriefing procedure is used, researchers can be confident that even rich false memories can be substantially reduced and, crucially, belief in the occurrence of the event can be almost entirely removed. The study also suggests a route through which false memories implanted in real-world settings (e.g., during psychotherapy or as a result of police questioning) might be reversed. The present study does not offer a direct corollary with these situations: in a research setting, we can state with a high degree of confidence that the fabricated event did not happen, and we can point to its precise provenance. This may not be the case for real-word memories. Nevertheless, where there is good evidence that the event in question did not really occur, a dehoaxing procedure in which individuals are educated about the nature of memory reconstruction and the mechanisms by which a false memory may be produced might be useful in reducing belief in the fictitious event.

### Supplementary Information

Below is the link to the electronic supplementary material.

Supplementary file1 (DOCX 34 KB)
