# Supplementary material for: Unringing the bell: Successful debriefing following a rich false memory study
Source: Mem Cognit. 2024 Jan 29;52(5):1079–92. doi: 10.3758/s13421-024-01524-9 (PMC11315748; doi:10.3758/s13421-024-01524-9)
Supplement: Supplementary file 1 — Supplementary file1 (DOCX 34 KB) [file 13421_2024_1524_MOESM1_ESM.docx]

**Unringing the bell: Successful debriefing following a rich false memory study**

**Supplemental materials**

***Table S-1***

*Preregistered analysis: Results of a logistic regression assessing the effects of phenomenological features on the presence or absence of a persistent false memory or false belief, assessed immediately after debriefing^1^*

|  | **B** | **S.E.** | **Wald** | **df** | **p** | **Exp(B)** | **95% C.I. for Exp(B)** | |
| --- | --- | --- | --- | --- | --- | --- | --- | --- |
|  |  |  |  |  |  |  | **Lower** | **Upper** |
| *False memory* |  |  |  |  |  |  |  |  |
| Clarity | 1.15 | 0.41 | 7.75 | 1 | 0.005* | 3.16 | 1.41 | 7.12 |
| Confidence in future memory | 0.14 | 0.22 | 0.40 | 1 | 0.53 | 1.15 | 0.74 | 1.78 |
| Sound | 0.07 | 0.21 | 0.11 | 1 | 0.74 | 1.07 | 0.71 | 1.63 |
| Evokes a feeling/reaction | 0.15 | 0.19 | 0.58 | 1 | 0.45 | 1.16 | 0.79 | 1.69 |
| Triggers memories | -0.31 | 0.16 | 3.99 | 1 | 0.05 | 0.73 | 0.54 | 0.99 |
| Ease of access | -0.65 | 0.38 | 2.90 | 1 | 0.09 | 0.52 | 0.25 | 1.10 |
| Constant | -3.64 | 1.04 | 12.19 | 1 | 0.00 | 0.03 |  |  |
|  |  |  |  |  |  |  |  |  |
| *False memory or false belief* | | |  |  |  |  |  |  |
| Clarity | 0.81 | 0.34 | 5.82 | 1 | 0.02* | 2.26 | 1.17 | 4.36 |
| Confidence in future memory | 0.18 | 0.19 | 0.97 | 1 | 0.33 | 1.20 | 0.83 | 1.74 |
| Sound | 0.10 | 0.18 | 0.27 | 1 | 0.60 | 1.10 | 0.77 | 1.58 |
| Evokes a feeling/reaction | 0.04 | 0.16 | 0.07 | 1 | 0.79 | 1.04 | 0.76 | 1.43 |
| Triggers memories | -0.16 | 0.12 | 1.84 | 1 | 0.18 | 0.85 | 0.68 | 1.07 |
| Ease of access | -0.48 | 0.33 | 2.05 | 1 | 0.15 | 0.62 | 0.33 | 1.19 |
| Constant | -3.00 | 0.83 | 13.18 | 1 | 0.00 | 0.05 |  |  |

^1^Due to multicollinearity between predictor variables, these analyses are not considered reliable. Please see main manuscript for alternative analyses.

*significant at p < .05

***Table S-2***

*Preregistered analysis: Results of a logistic regression assessing the effects of phenomenological features on presence or absence of a persistent false memory or false belief, assessed in the follow-up survey^1^*

|  | **B** | **S.E.** | **Wald** | **df** | **p** | **Exp(B)** | **95% C.I. for Exp(B)** | |
| --- | --- | --- | --- | --- | --- | --- | --- | --- |
|  |  |  |  |  |  |  | **Lower** | **Upper** |
| *False memory* |  |  |  |  |  |  |  |  |
| Clarity | 0.68 | 0.53 | 1.67 | 1 | 0.20 | 1.98 | 0.70 | 5.59 |
| Confidence in future memory | -0.04 | 0.33 | 0.01 | 1 | 0.92 | 0.97 | 0.50 | 1.86 |
| Sound | -0.11 | 0.27 | 0.15 | 1 | 0.70 | 0.90 | 0.53 | 1.54 |
| Evokes a feeling/reaction | -0.11 | 0.31 | 0.14 | 1 | 0.71 | 0.89 | 0.49 | 1.64 |
| Triggers memories | 0.01 | 0.21 | 0.00 | 1 | 0.96 | 1.01 | 0.67 | 1.54 |
| Ease of access | 0.22 | 0.53 | 0.17 | 1 | 0.68 | 1.25 | 0.44 | 3.53 |
| Constant | -4.74 | 1.58 | 9.01 | 1 | 0.00 | 0.01 |  |  |
|  |  |  |  |  |  |  |  |  |
| *False memory or false belief* | | |  |  |  |  |  |  |
| Clarity | 0.61 | 0.42 | 2.18 | 1 | 0.14 | 1.85 | 0.82 | 4.18 |
| Confidence | -0.23 | 0.28 | 0.71 | 1 | 0.40 | 0.79 | 0.46 | 1.36 |
| Sound | -0.16 | 0.24 | 0.43 | 1 | 0.51 | 0.86 | 0.54 | 1.36 |
| Evokes a feeling/reaction | 0.03 | 0.17 | 0.02 | 1 | 0.89 | 1.03 | 0.74 | 1.43 |
| Triggers memories | 0.03 | 0.13 | 0.05 | 1 | 0.83 | 1.03 | 0.80 | 1.32 |
| Ease of access | -0.02 | 0.37 | 0.00 | 1 | 0.95 | 0.98 | 0.47 | 2.03 |
| Constant | -2.87 | 0.94 | 9.37 | 1 | 0.002 | 0.06 |  |  |

^1^Due to multicollinearity between predictor variables, these analyses are not considered reliable. Please see main manuscript for alternative analyses.

***Table S-3***

*Descriptive statistics and univariate comparison of phenomenological feature ratings between those who reported a memory (or belief) and those who did not, when assessed prior to debriefing at the end of Interview 2*

|  | **False memory** | | |  | **No false memory** | | |  |  |
| --- | --- | --- | --- | --- | --- | --- | --- | --- | --- |
|  | **N** | **M** | **SD** |  | **N** | **M** | **SD** |  | **Univariate effect** |
| Clarity | 17 | 4.82 | 1.85 |  | 106 | 1.72 | 1.19 |  | F(1, 121) = 84.7, p < .001, η_p_^2^ = 0.41* |
| Confidence in future memory | 17 | 3.88 | 2.34 |  | 106 | 1.93 | 1.42 |  | F(1, 121) = 22.56, p < .001, η_p_^2^ = 0.16* |
| Sound | 17 | 2.88 | 1.87 |  | 106 | 1.65 | 1.47 |  | F(1, 121) = 9.54, p = .002, η_p_^2^ = 0.07* |
| Evokes a feeling/reaction | 17 | 6.06 | 1.85 |  | 106 | 3.16 | 2.26 |  | F(1, 121) = 25.1, p < .001, η_p_^2^ = 0.17* |
| Triggers memories | 17 | 4.29 | 2.91 |  | 106 | 5.33 | 2.97 |  | F(1, 121) = 1.79, p = .18, η_p_^2^ = 0.02 |
| Ease of access | 17 | 4.53 | 2.24 |  | 106 | 1.54 | 1.24 |  | F(1, 121) = 65.4, p < .001, η_p_^2^ = 0.35* |
|  | **False memory OR belief** | | |  | **Neither false memory nor belief** | | |  |  |
|  | **N** | **M** | **SD** |  | **N** | **M** | **SD** |  | **Univariate effect** |
| Clarity | 81 | 2.47 | 1.90 |  | 42 | 1.52 | 0.86 |  | F(1, 121) = 9.39, p = 0.003, η_p_^2^ = 0.07* |
| Confidence in future memory | 81 | 2.46 | 1.89 |  | 42 | 1.71 | 1.13 |  | F(1, 121) = 5.45, p = 0.02, η_p_^2^ = 0.04* |
| Sound | 81 | 2.06 | 1.78 |  | 42 | 1.36 | 0.93 |  | F(1, 121) = 5.72, p = 0.02, η_p_^2^ = 0.04* |
| Evokes a feeling/reaction | 81 | 3.79 | 2.49 |  | 42 | 3.12 | 2.26 |  | F(1, 121) = 2.14, p = 0.15, η_p_^2^ = 0.02 |
| Triggers memories | 81 | 5.19 | 2.95 |  | 42 | 5.19 | 3.06 |  | F(1, 121) = 0, p = 0.99, η_p_^2^ = 0 |
| Ease of access | 81 | 2.30 | 2.03 |  | 42 | 1.29 | 0.64 |  | F(1, 121) = 9.89, p = 0.002, η_p_^2^ = 0.08* |

*significant at p < .05

***Table S-4***

*Descriptive statistics and univariate comparison of phenomenological feature ratings between those who reported a memory (or belief) and those who did not, when assessed just after debriefing*

|  | **False memory** | | | |  | **No false memory** | | | |  |  | |
| --- | --- | --- | --- | --- | --- | --- | --- | --- | --- | --- | --- | --- |
|  | | **N** | **M** | **SD** | |  | **N** | **M** | **SD** | |  | **Univariate effect** |
| Clarity | | 13 | 4.23 | 1.64 | |  | 105 | 1.94 | 1.53 | |  | F(1, 116) = 25.45, p < .001, η_p_^2^ = 0.18* |
| Confidence in future memory | | 13 | 3.46 | 1.98 | |  | 105 | 2.10 | 1.64 | |  | F(1, 116) = 7.68, p = .007, η_p_^2^ = 0.06* |
| Sound | | 13 | 3.23 | 1.96 | |  | 105 | 1.69 | 1.48 | |  | F(1, 116) = 11.74, p = .001, η_p_^2^ = 0.09* |
| Evokes a feeling/reaction | | 13 | 4.85 | 1.73 | |  | 105 | 3.48 | 2.47 | |  | F(1, 116) = 3.76, p = .055, η_p_^2^ = 0.03 |
| Triggers memories | | 13 | 3.77 | 2.17 | |  | 105 | 5.33 | 3.05 | |  | F(1, 116) = 3.2, p = .08, η_p_^2^ = 0.03 |
| Ease of access | | 13 | 3.62 | 2.06 | |  | 105 | 1.79 | 1.64 | |  | F(1, 116) = 13.52, p < .001, η_p_^2^ = 0.1* |
|  | | **False memory OR belief** | | | |  | **Neither false memory nor belief** | | | |  |  |
|  | | **N** | **M** | **SD** | |  | **N** | **M** | **SD** | |  | **Univariate effect** |
| Clarity | | 17 | 3.76 | 1.786 | |  | 101 | 1.93 | 1.538 | |  | F(1, 116) = 19.74, p < .001, η_p_^2^ = 0.14* |
| Confidence in future memory | | 17 | 3.41 | 1.938 | |  | 101 | 2.05 | 1.615 | |  | F(1, 116) = 9.76, p = .002, η_p_^2^ = 0.08* |
| Sound | | 17 | 2.94 | 1.886 | |  | 101 | 1.67 | 1.484 | |  | F(1, 116) = 9.79, p = .002, η_p_^2^ = 0.08* |
| Evokes a feeling/reaction | | 17 | 4.53 | 1.972 | |  | 101 | 3.48 | 2.476 | |  | F(1, 116) = 2.78, p = .10, η_p_^2^ = 0.02 |
| Triggers memories | | 17 | 4.35 | 2.317 | |  | 101 | 5.3 | 3.09 | |  | F(1, 116) = 1.45, p = .23, η_p_^2^ = 0.01 |
| Ease of access | | 17 | 3.24 | 2.016 | |  | 101 | 1.78 | 1.653 | |  | F(1, 116) = 10.54, p = .002, η_p_^2^ = 0.08* |

*significant at p < .05

***Table S-5***

*Descriptive statistics and univariate comparison of phenomenological feature ratings between those who reported a memory (or belief) and those who did not, when assessed in the follow-up survey*

|  | **False memory** | | | |  | **No false memory** | | | |  |  | |
| --- | --- | --- | --- | --- | --- | --- | --- | --- | --- | --- | --- | --- |
|  | | **N** | **M** | **SD** | |  | **N** | **M** | **SD** | |  | **Univariate effect** |
| Clarity | | 6 | 4.67 | 2.422 | |  | 95 | 1.93 | 1.386 | |  | F(1, 99) = 19.99, p < .001, η_p_^2^ = 0.17* |
| Confidence in future memory | | 6 | 3.33 | 1.966 | |  | 95 | 2.05 | 1.607 | |  | F(1, 99) = 3.5, p = .06, η_p_^2^ = 0.03 |
| Sound | | 6 | 3 | 2.53 | |  | 95 | 1.73 | 1.519 | |  | F(1, 99) = 3.64, p = .06, η_p_^2^ = 0.03 |
| Evokes a feeling/reaction | | 6 | 4.83 | 2.137 | |  | 95 | 3.48 | 2.369 | |  | F(1, 99) = 1.85, p = .18, η_p_^2^ = 0.02 |
| Triggers memories | | 6 | 4.33 | 1.966 | |  | 95 | 5.16 | 3.012 | |  | F(1, 99) = 0.44, p = .51, η_p_^2^ = 0.004 |
| Ease of access | | 6 | 4.33 | 2.503 | |  | 95 | 1.76 | 1.449 | |  | F(1, 99) = 16.2, p < .001, η_p_^2^ = 0.14* |
|  | | **False memory OR belief** | | | |  | **Neither false memory nor belief** | | | |  |  |
|  | | **N** | **M** | **SD** | |  | **N** | **M** | **SD** | |  | **Univariate effect** |
| Clarity | | 12 | 3.17 | 2.329 | |  | 89 | 1.94 | 1.417 | |  | F(1, 99) = 6.62, p = .01, η_p_^2^ = 0.06* |
| Confidence in future memory | | 12 | 2.42 | 1.73 | |  | 89 | 2.09 | 1.642 | |  | F(1, 99) = 0.41, p = .52, η_p_^2^ = 0.004 |
| Sound | | 12 | 2 | 2 | |  | 89 | 1.78 | 1.558 | |  | F(1, 99) = 0.2, p = .65, η_p_^2^ = 0.002 |
| Evokes a feeling/reaction | | 12 | 4.25 | 2.05 | |  | 89 | 3.47 | 2.403 | |  | F(1, 99) = 1.14, p = .29, η_p_^2^ = 0.01 |
| Triggers memories | | 12 | 5 | 2.089 | |  | 89 | 5.12 | 3.067 | |  | F(1, 99) = 0.02, p = .89, η_p_^2^ = .001 |
| Ease of access | | 12 | 2.83 | 2.368 | |  | 89 | 1.79 | 1.481 | |  | F(1, 99) = 4.51, p = .04, η_p_^2^ = 0.04* |

*significant at p < .05
